# Whole-Body Bone Scan for Detecting Bone Metastasis in the Prostate-Specific Membrane Antigen Positron Emission Tomography Era: A Retrospective Cohort Study of Post-Radical Prostatectomy Prostate Cancer Patients

**DOI:** 10.22038/aojnmb.2025.82544.1582

**Published:** 2025

**Authors:** Chanikarn Poenateetai, Achiraya Teyateeti, Pawana Pusuwan, Ajalaya Teyateeti

**Affiliations:** 1 Division of Nuclear Medicine, Department of Radiology, Faculty of Medicine Siriraj Hospital, Mahidol University, Bangkok, Thailand; 2 Division of Radiation Oncology, Department of Radiology, Faculty of Medicine Siriraj Hospital, Mahidol University, Bangkok, Thailand

**Keywords:** Prostate cancer, Radical prostatectomy Biochemical recurrence, Bone metastasis, Bone scan

## Abstract

**Objective(s):**

To determine the detection rate of bone metastasis on bone scan of prostate cancer patients with rising serum prostate-specific antigen (PSA) following radical prostatectomy (RP) and to identify the predictive factors associated with bone metastasis.

**Methods:**

A study was conducted in 120 patients with rising serum PSA after RP. The data collected were pre and post-RP clinical parameters, including a trigger PSA (tPSA) level that prompted the treating physician to request a bone scan and PSA doubling time (PSADT). Bone scans were classified as positive or negative in conjunction with follow-up imaging and clinical information.

**Results:**

Of 120 bone scans, 6 (5%) were positive and 114 (95%) were negative for bone metastasis. In the median tPSA ranges of <0.5, 0.5-1.0, and >1.0 ng/mL, scan positivity was 2.1%, 6.3%, and 30%, respectively. Patients with positive scans showed higher tPSA (1.228 vs 0.256 ng/mL; p=0.003) and shorter PSADT (3.5 vs 12.2 months; p=0.005) than those with negative scans. The most significant predictors of a positive bone scan were tPSA (>1 vs ≤1 ng/mL; OR 15.286, 95% CI 2.594-90.064, p=0.003) and PSADT (<6 vs ≥6 months; OR 17.333, 95% CI 1.618-185.646, p=0.018).

**Conclusion:**

The detection rate of bone metastasis on bone scans in post-RP recurrent prostate cancer patients is only 5%, but the probability is much higher with tPSA >1 ng/mL and PSADT <6 months. Given its wide accessibility in Thailand, a bone scan should remain the preferred screening test for bone metastasis, with expected positive results in patients with high or rapidly rising PSA levels.

## Introduction

 Radical prostatectomy (RP) is one of the most established therapies for prostate cancer that provides excellent control for clinically localized disease (1-3). However, almost a third of prostate cancer patients will ultimately develop tumor recurrence within 10 years of radical prostatectomy (4-9). 

 These recurrences can be classified as either local or systemic and often manifest as a detectable or rising serum prostate- specific antigen (PSA), termed as biochemical recurrence (BCR) (10).

 In recent years, molecular imaging with prostate-specific membrane antigen-positron emission tomography/computed tomography 

(PSMA-PET/CT) has shown promising results for detecting loco-regional and distant metastases that clearly surpass conventional imaging such as abdominal CT, magnetic resonance imaging (MRI) of prostate gland and technetium-^99^m methylene diphosphonate (^99m^Tc-MDP) bone scan in terms of sensitivity and specificity (11-14). The European Association of Urology (EAU) Guidelines now recommend PSMA PET/CT as the imaging modality of choice in patients with PSA relapse after radical treatment (15). Nevertheless, the associated logistical challenges, high cost per procedure, and reimbursement issues have rendered PSMA PET/CT inaccessible to many patients in Thailand. In this context, bone scan is still widely accepted as one of the standard diagnostic tools for investigating bone metastasis, although the probability of a positive scan is relatively low.

 Various authors in the last couple of decades have reported conflicting data regarding the detection rates of bone scan in patients with BCR, although most seem to agree that PSA level before bone scan, PSA velocity (PSAV), and PSA doubling time (PSADT) are strong predictors of bone scan positivity (10, 11, 16-22). 

 Nevertheless, in the current era of ultrasensitive PSA testing and PSMA-PET/CT, where the focus of management is shifting towards early detection and treatment, the role of bone scan remains unclear.

 In this present study, we aimed to evaluate the detection rate of bone metastasis on whole body bone scan of prostate cancer patients with rising serum PSA following RP. Furthermore, our secondary objective was to identify factors associated with bone scan positivity, which may have useful clinical implications regarding the patient selection.

## Methods

Following approval by the institutional review board, which waived the requirement for patient consent, we performed a retrospective review of all patients who underwent RP (±pelvic lymph node dissection) and subsequently had detectable or rising serum PSA that were investigated with bone scan at the Division of Nuclear Medicine, Department of Radiology, Faculty of Medicine Siriraj Hospital between January 2018 and April 2022. Patients with bone or other distant metastasis at initial diagnosis, who received adjuvant radiotherapy or androgen-deprivation therapy (ADT), or those without clinical and/or radiological follow-up were not enrolled in this study.

 Enrolled patients were staged based on pathological assessment of the tumor specimen according to the 8th edition American Joint Committee on Cancer (AJCC) Staging System and histologic grading was based on the Gleason grading system (23). Post-operative surveillance consisted of medical history, physical examination, serum PSA levels, and imaging tests including bone scan and other modalities, at the discretion of the treating physician. The trigger PSA (tPSA) was defined as the PSA level that prompted the treating physician to request a bone scan. PSA kinetics, i.e., PSADT and PSAV (in months) were calculated according to the previously described method (10, 24). Eligible subjects for this calculation were those with all PSA values greater than 0.1 ng/mL and at least three PSA values taken at intervals of 3 months or more.

 Bone scan was performed after intravenous administration of 20 mCi ±10% of ^99m^Tc-MDP. Planar whole-body imaging in anterior and posterior projections were obtained approximately 3 hours post-injection with additional spot views or single photon emission computed tomography (SPECT)/CT images, if indicated. Our 5 GE discovery gamma cameras – 4 conventional detector (670 series and 870 series) and 1 solid state detector – were comparable in terms of image quality. All bone scans were reviewed independently by two nuclear medicine physicians (3 and 14 years of experience) in conjunction with supporting evidence, i.e., follow-up imaging, clinical data and treatments. Equivocal studies and any disagreements were resolved by consensus after consulting with a third reviewer (a senior nuclear medicine physician with over 30 years of experience).

 Baseline characteristics were collected, including patient age at time of diagnosis, initial PSA (iPSA), Gleason score (GS), pathological staging with tumor staging (pT) and lymph node (LN) staging (pN), surgical margin (SM) status, extra-prostatic extension (EPE), and seminal vesicle invasion (SVI). Additionally, PSA characteristics and kinetics were gathered, comprising of post-RP PSA nadir, PSAV, PSADT, tPSA, and time to PSA recurrence. The ultrasensitive PSA assay was used, with an undetectable PSA level defined as less than 0.003 ng/mL. Findings and results from bone scans, including the number and sites of metastatic lesions, as well as those from other imaging modalities performed within a 3-month period for detecting recurrent disease, were documented.

 Descriptive statistics were employed to characterize patient demographic data and bone scan results. Categorical variables were presented as numbers and percentages, while continuous variables were presented as medians with ranges. Group comparisons were conducted using the χ² test for categorical variables and the Student t test for continuous variables. Predictive factors associated with bone scan positivity were analyzed using a logistic regression model and reported as odds ratios (OR) with 95% confidence intervals (CI). Statistical analyses were performed using IBM SPSS Statistics for Windows, Version 21.0 (IBM Corp., Armonk, NY). A p-value of less than 0.05 was considered statistically significant.

## Results

### Patient characteristics

 One hundred twenty patients with BCR after RP were identified. None had received additional treatment besides RP. Median iPSA was 10.55 ng/mL (range 1.79-61.95). Eighty-six patients (71.7%) had GS of 6-7, whereas the remaining thirty-four patients (28.3%) had GS of 8-10. EPE and SVI were not present in the majority of patients (58.3% and 88.3%, respectively). Fifty-seven patients (47.5%) showed a positive SM. Only 1 patient (0.8%) had LN involvement. Table 1 outlines the clinical and pathological characteristics of the study population, both overall and categorized by bone scan positivity.

**Table 1:** Clinical and pathological characteristics of the study population

**Characteristics**	**All** **(n=120)**	**Positive bone scan** **(n=6)**	**Negative bone scan** **(n=114)**	**P**
Age at diagnosis (years), median (range)	67 (50-81)	65 (51-72)	67 (50-81)	0.455
iPSA (ng/mL)* Median (range) Level, n (%) < 10 10-20 > 20	10.55 (1.79-61.95) 51 (43.2) 45 (38.1) 22 (18.6)	10.46 (4.8-25) 2 (40) 2 (40) 1 (20)	10.63 (1.79-61.95) 49 (43.4) 43 (38.1) 21 (18.6)	0.947 1.000
GS, n (%) GS 6-7 GS 8-10	86 (71.7) 34 (28.3)	2 (33.3) 4 (66.7)	84 (73.7) 30 (26.3)	0.053
pT, n (%) T2 T3-T4	63 (52.5) 57 (47.5)	4 (66.7) 2 (33.3)	59 (51.8) 55 (48.2)	0.682
pN, n (%) N0 N1	119 (99.2) 1 (0.8)	6 (100) 0	113 (99.1) 1 (0.9)	1.000
EPE, n (%) Absence Presence	70 (58.3) 50 (41.7)	4 (66.7) 2 (33.3)	66 (57.9) 48 (42.1)	1.000
SM, n (%) Absence Presence	57 (47.5) 63 (52.5)	3 (50) 3 (50)	54 (47.4) 60 (52.6)	1.000
SVI Absence Presence	106 (88.3) 14 (11.7)	5 (83.3) 1 (16.7)	101 (88.6) 13 (11.4)	0.533
Post-RP PSA nadir (ng/mL) Median, range Level, n (%) Undetectable+ Detectable	0.0045 (0-26.54) 59 (49.2) 61 (50.8)	0.0366 (0.0030-26.54) 1 (16.7) 5 (83.3)	0.0030 (0-6.8) 58 (50.9) 56 (49.1)	0.037 0.207
tPSA (ng/mL) Median, range Level, n (%) < 0.5 0.5-1.0 > 1.0	0.26 (0.009-26.54) 94 (78.3) 16 (13.3) 10 (8.3)	1.228 (0.258-26.54) 2 (33.3) 1 (16.7) 3 (50)	0.256 (0.009-6.080) 92 (80.7) 15 (13.2) 7 (6.1)	0.003 0.006
Time from RP to tPSA (month), median (range)	32.48 (1.51-213.19)	12.81 (1.51-82)	33.61 (2.63-213.19)	0.069
PSADT# (months) Median (range) Level, n (%) < 6 ≥ 6	11.46 (0.51-120.88) 12 (18.5) 53 (81.5)	3.515 (0.51-6.72) 3 (75) 1 (25)	12.2 (1.96-120.88) 9 (14.8) 52 (85.2)	0.005 0.018
PSAV# (ng/mL/month), median (range)	0.01 (0-2.99)	0.085 (0.03-2.99)	0.010 (0-0.12)	0.002

^*^iPSA (n=118); 2 patients with missing iPSA values

+Undetectable PSA was defined as <0.003 ng/mL

#PSADT and PSAV calculation was not available in 55 patients due to the following reasons; 15 patients – all PSA values <0.1, 40 patients – less than 3 eligible PSA values

### Detection of bone metastasis

 Of the 120 bone scans, 6 (5%) were positive and 114 (95%) were negative for bone metastasis. All six patients with positive bone scans had oligometastasis, which was defined as having fewer than 5 bone lesions (Table 2). The positivity rate of bone scans, stratified by median tPSA ranges, was 2.1% for less than 0.5 ng/mL, 6.3% for 0.5-1.0 ng/mL, and 30% for above 1.0 ng/mL (Table 3).

 Patients with positive bone scans had significantly higher post-RP PSA nadir (0.0366 vs 0.0030 ng/mL; p=0.037) and tPSA (1.228 vs 0.256 ng/mL; p=0.003) than patients with negative bone scans. Age at time of diagnosis, iPSA, pathological stage, SM, EPE and SVI status were not significantly different between patients with positive and negative bone scans. Men with positive scans tended to have higher GS of 8-10 (66.7 vs 26.3%; p=0.053) and shorter median time from RP to tPSA (12.81 vs 33.61 months; p=0.069) compared to men with negative scans, although the differences were not statistically significant.

 Sixty-five of 120 patients qualified for analysis of PSADT and PSAV. Of the remaining 55 ineligible patients, 15 had serial PSA values below 0.1 ng/mL and 40 had less than 3 eligible PSA values. Patients with positive bone scans had much shorter PSADT (3.515 vs 12.2 months; p=0.005) as well as higher PSAV (0.085 vs 0.010 ng/mL/month; p=0.002) than those who had negative bone scans.

**Table 2:** Characteristics and imaging findings of patients with positive bone scans

	**GS**	**pT**	**pN**	Post-op PSA nadir*	**tPSA** ^*^	**Time from RP to tPSA** ^+^	**PSADT** ^+^	**Imaging findings** ^#^			
								**Imaging**	**PB**	**PLN**	**B (BS** ^$^ **/PSMA)**
1	9(4+5)	T2	N0	0.008	0.258	8.38	-	MRI, PSMA	-	-	1 / 2
2	7(3+4)	T2	N0	0.050	0.40	14.32	4.61	MRI	-	-	2
3	7(3+4)	T2	N0	0.023	0.676	11.30	2.42	MRI, PSMA	+	+	1 / 1
4	9(4+5)	T3a	N0	<0.003	1.780	82.0	6.72	MRI, PSMA	+	-	3 / 4
5	8(4+4)	T2	N0	0.120	9.460	24.21	0.51	MRI	-	-	2
6	9(4+5)	T3b	N0	26.54	26.54	1.51	-	F-18 choline	-	-	2

^*^tPSA – trigger PSA (ng/mL); +time (month); # imaging findings PB - prostate bed, PLN – pelvic lymph node metastasis, B - bone metastasis (number of lesion on bone scan/F-18 PSMA PET/CT)

$locations of metastatic lesions on bone scans – patient no.1 L2 vertebra, no. 2 left 5th and 6thribs, no. 3 left iliac bone, no. 4 left sacroiliac joint, left acetabulum and right superior pubic ramus, no. 5 T9 vertebra and left iliac bone and no. 6 right acetabulum and right pubic bone

**Table 3:** tPSA level and positivity of bone scans

tPSA level (ng/mL)	Total scan (n=120)	Positive scan (n=6)	% Positive
**<0.5**	94	2	2.1
**0.5–1.0**	16	1	6.3
**>1.0**	10	3	30

 In logistic regression analysis, tPSA (>1 vs ≤1 ng/mL; OR 15.286, 95% CI 2.594-90.064, p=0.003) and PSADT (<6 vs ≥6 months; OR 17.333, 95% CI 1.618-185.646, p=0.018) were significant predictors of a positive bone scan (Table 4).

**Table 4:** Relationship of variables with bone scan outcome in logistic regression analysis

**Factors**	**OR (95%CI)**	**P**
**GS:** 6-7 vs 8-10	5.6 (0.975-32.156)	0.053
**Post-RP PSA nadir:** Undetectable vs detectable	5.179 (0.586-45.729)	0.139
**tPSA:** ≤1.0 vs >1.0 ng/mL	15.286 (2.594-90.064)	0.003
**Time from RP to tPSA:** <12 vs ≥12 months	4.429 (0.835-23.499)	0.081
**PSADT:** ≥6 vs <6 months	17.333 (1.618-185.646)	0.018

### Additional imaging findings

**Figure 1:**
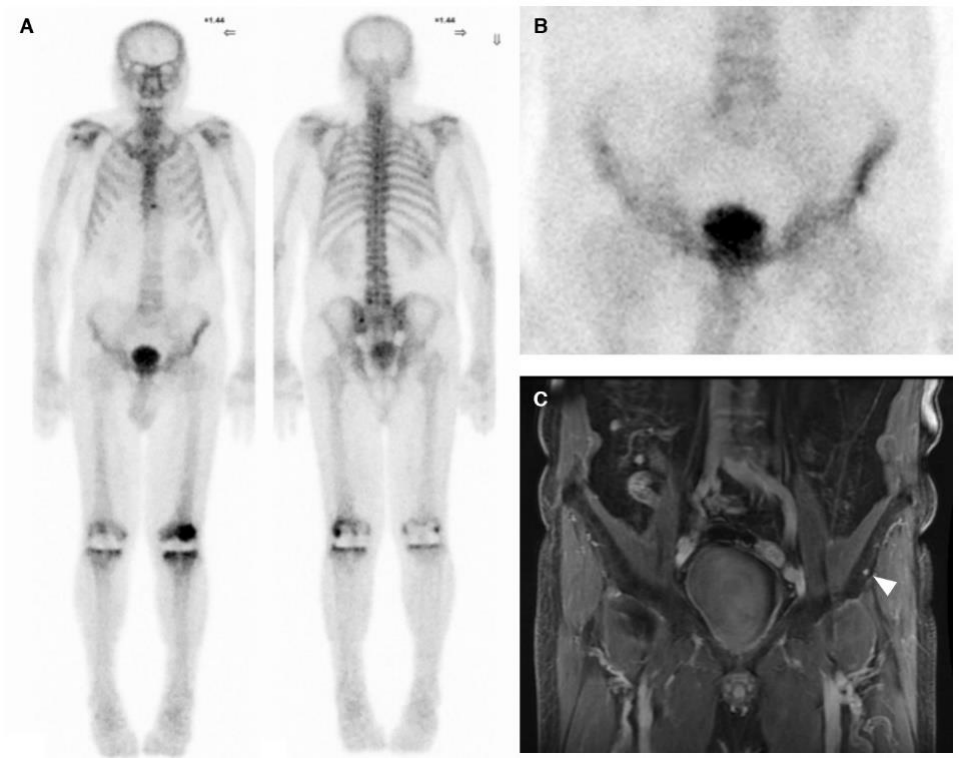
A 78-year-old patient-year-old patient (Gleason score 7(3+4), pT3aN0M0) with BCR (tPSA=0.226 ng/mL) almost 3 years after laparoscopic radical prostatectomy. (**A**) Bone scan showed no definite evidence of bone metastasis. Areas of increased uptake at the sternum, L5 vertebra, left sacroiliac region, and both knees were most likely due to degenerative or inflammatory processes. (**B**) Spot view of the pelvis was unremarkable. Prominent uptake at the left iliac spine, relative to the right side, was likely due to asymmetrical positioning. (**C**) MRI scan of the abdomen revealed a suspicious 0.5-cm lesion at the left iliac bone that was not previously detected on bone scan (**white arrow head**). This lesion was considered non-specific, and treatment was administered as if no bone metastasis was present

 The diagnosis of bone metastasis was confirmed by at least one other imaging modality – MRI and/or PET/CT using Flourine-18 (^18^F) PSMA-1007 within 3 months of bone scan in all of the 6 patients with positive bone scans. One-hundred of 114 patients from the negative scan group had received additional conventional imaging (whole abdominal CT and/or prostate MRI), 83 (83%) of which were negative for abnormalities. Additional imaging of the remaining 17 patients revealed suspicious findings at the prostate bed (14%), intra-abdominal lymphadenopathy (3%), and bone (2%) (Figure 1).

 Six patients received further ^18^F-PSMA PET/CT imaging within 3 months of their negative bone scan results. Amongst these patients, ^18^F-PSMA PET/CT revealed suspicions for local recurrence at the prostate bed in 2 patients (33.3%) and intra-abdominal lymphadenopathy in 1 patient (16.7%), all of which were not previously detected on MRI. Equivocal bone lesions resembling non-specific bone uptake were recorded in 2 of the 6 patients (33.3%).

## Discussion

Recent studies have shown excellent long-term survival outcomes in patients that received early salvage radiotherapy as treatment for PSA relapse following RP, such that currents trends are shifting towards very early salvage radiotherapy in patients with any detectable PSA level rather than waiting until the traditional PSA threshold of 0.5 ng/mL (25-33). This occurrence is further enhanced by the widespread application of ultrasensitive PSA assays, which are capable of detecting PSA levels as low as 0.001 ng/mL (34). As a result, early detection of local recurrence or metastatic disease is of the utmost importance for directing salvage therapy.

 In Thailand, the radiologic investigation of patients with BCR after RP has traditionally included both a ^99^mTc-MDP bone scan and abdominopelvic CT/MRI. However, there is a lack of consensus regarding when patients should be screened for metastasis, as well as no clear PSA cut-off that would prompt such investigations. With the purpose of guiding screening practices, we tried to more precisely define the clinical utility of bone scanning in patients with biochemically recurrent prostate cancer and identified variables that would have significantly impacted the outcome of these scans.

 In the present study, we found that the metastasis detection rate of bone scan in patients with BCR following RP was approximately 5%. These results were in line with prior studies by Cher et al. (16) and Moreira et al. (22) that have reported similar detection rates in hormone-naïve subjects of 4.1% and 6%, respectively.

 We found that baseline characteristics (i.e., iPSA, tumor staging, LN involvement, SM status, EPE and SVI) had no effect on bone scan positivity. Although a large proportion of patients with positive bone scans had higher GS, logistic regression analysis failed to demonstrate a significant correlation between GS and bone scan outcome (p=0.053). In contrast, post-RP PSA characteristics were more likely to influence bone scan outcome, as patients with positive bone scans showed higher median post-RP PSA nadir (p=0.037), higher median tPSA (p=0.003), shorter median PSADT (p=0.005), as well as higher median PSAV (p=0.002) than those with negative bone scans. Our analysis suggests that both tPSA levels greater than 1 ng/mL (p=0.003) and PSADT of less than 6 months (p=0.018) were significant predictors of a positive bone scan. It is of note that although multiple previous studies have confirmed the association between tPSA and bone scan positivity (11, 16-22), only two have arrived to a similar conclusion with PSADT (11, 22). Our data supports the hypothesis that the patterns of biochemical failure following RP, rather than initial pathologic parameters, are the more significant predictors of metastatic disease.

 In our practice, we noted that a large number of bone scans were requested in patients with relatively low serum PSA values, hence the lower tPSA values reported in our cohort (median tPSA 0.26 ng/mL) as opposed to prior studies (tPSA 5-10 ng/mL or more). The probability of a positive bone scan remained less than 3% (3 of 110 patients) until patients reached a tPSA level of greater than 1 ng/mL, at which there is 30% (3 of 10 patients) chance of a positive bone scan. As a result, a serum PSA level of 1 ng/mL may be the optimal PSA cut-off for bone metastasis screening in patients with BCR.

 We also found that in men with PSADT less than 6 months the incidence of a positive bone scan was 25% (3 of 12 patients) as compared to less than 2% (1 of 53 patients) in those with PSADT of more than 6 months. These results may imply that the utility of bone scan is rather limited in patients with slow PSA progression.

 Over 90% of patients in our cohort had a tPSA level less than 1.0 ng/mL. To the best of our knowledge, this is one of very few studies to report bone scan findings at a median tPSA level as low as 0.26 ng/mL. At such a low tPSA level (≤0.5 ng/mL), existing literature mostly mentions PSMA PET/CT. In a prospective study on the application of ^18^F-PSMA-1007 PET/MR in early BCR prostate cancer patients (defined as PSA level ≤0.5 ng/mL) with a comparable median tPSA level of 0.31 ng/mL, patients with PSMA-avid bone metastasis accounted for only 4.8% of all patients (3 of 62 patients) (35). 

 Notably, even with the ^18^F-PSMA-1007 PET/MR scan, the detection rate of bone metastasis is not much higher than that of bone scans (2.1% and 6.3% at PSA level <0.5 ng/mL and 0.5-1.0 ng/mL, respectively). These findings suggest that the incidence of bone metastasis at low PSA levels, particularly less than 0.5 ng/mL, is generally low across all diagnostic imaging modalities. Therefore, bone scans continue to be the preferred choice for bone metastatic screening in Thailand, as they are more accessible and cost-effective than PSMA PET/CT.

**Figure 2:**
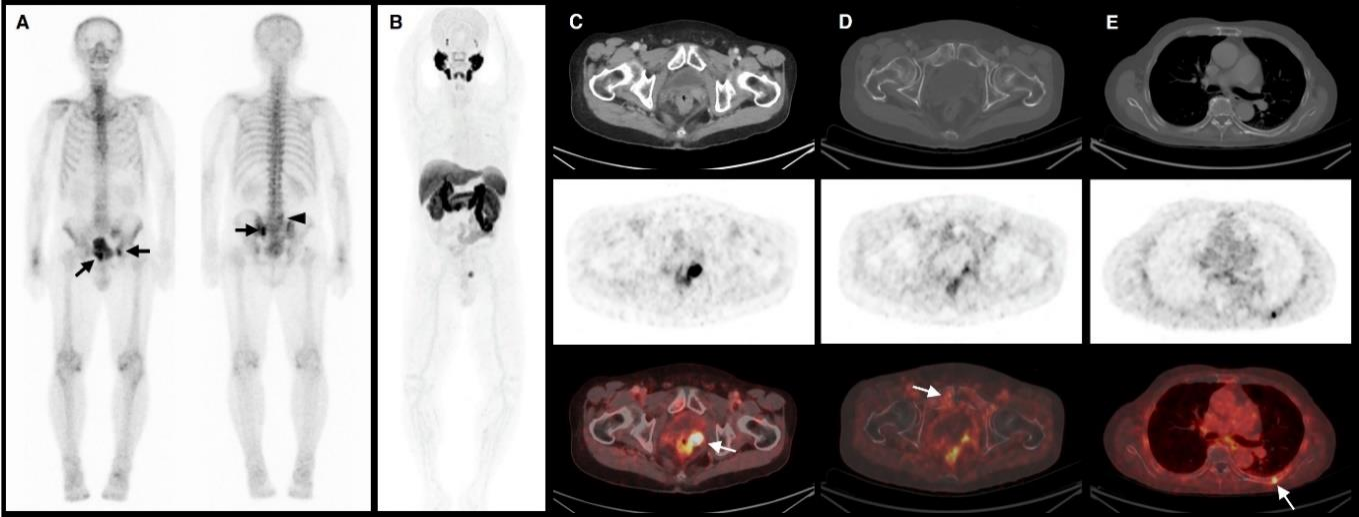
A 75-year-old patient (Gleason score 9(4+5), pT3aN0M0) with BCR (tPSA=1.78 ng/mL) almost 7 years after robotic-assisted laparoscopic radical prostatectomy. (**A**) Bone scan showed increased uptake at the right superior pubic ramus, left sacroiliac region, and left acetabulum (**black arrows**) that were most likely bone metastasis. Another area of increased uptake at the left side of L5 vertebra was likely due to degenerative change (**black arrow head**). (**B**) ^18^F-PSMA-1007 PET/CT scan performed on the same patient revealed (**C**) local recurrence at the prostate bed, (**D**) mildly PSMA-avid osteoblastic lesions at the right superior pubic ramus, (not shown) left sacroiliac region, and left acetabulum, that were consistent with bone scan findings and supported a diagnosis of bone metastasis. (**E**) Focal PSMA avidity at the left 7^th^ rib without CT correlate favored non-specific bone uptake

 Another particular interesting finding was that for two instances that bone scan and ^18^F-PSMA-1007 PET/CT were performed in the same patient, ^18^F-PSMA-1007 PET/CT was able to detect a greater number of suspicious bone lesions, demonstrating superior sensitivity to bone scan (patients no. 1 and 4 from Table 2). However, it should also be noted that for patients who had negative bone scan but positive ^18^F-PSMA-1007 PET/CT, most PSMA-avid lesions were mainly locoregional recurrences (15 of 17 patients). Almost all PSMA-avid bone lesions were non-specific uptake without associated osteolytic or osteoblastic lesion. Only one patient was found to have PSMA-avid osteoblastic bone lesions (patient no. 4 from Table 2) that was considered a true-positive result (Figure 2). Non-specific bone uptake on ^18^F-PSMA PET/CT is becoming an increasingly reported phenomenon and may lead to a false-positive diagnosis (36-38). This in turn, may result in overstaging and inappropriate treatment decisions. The European Society for Radiotherapy and Oncology (ESTRO) advises against recommending metastatic directed-therapy for bone lesions with only PSMA uptake and no radiological correlate on CT scan as they rarely represent metastasis (39). Caution should be taken when assessing bone uptake ^18^F-PSMA PET/CT given the potential diagnostic pitfall.

The main limitation of the present study is its retrospective nature. Therefore, the analysis might be prone to selection bias and missing information. We were not able to control when bone scans were conducted as clinical judgement varied between cases. In addition, PSA monitoring in patients after RP did not follow a protocol and were at the discretion of the treating physician. Over 45% of our study sample also had missing PSA kinetics. Another point to consider was that a portion of our study overlapped with the COVID-19 pandemic, during which the imposed societal restrictions severely disrupted prostate cancer surveillance across the country. The lock-down resulted in a dramatic decrease in the number of PSA requests, delayed radiologic investigations and may have a negative impact on treatment decisions, which further added noise and unwanted variability to our data. Finally, although bone scans were reinterpreted by a consensus of two nuclear medicine physicians, the lack of correlation with pathological diagnosis may lower the accuracy of our results.

 The strength of our study lies in its clinical applicability to current practices in Thailand, where bone scan and conventional imaging remain the standard diagnostic tools in metastatic work-up as they are readily accessible to the general population, cost-effective, simple to perform, and can qualify for reimbursement according to the public health coverage.

## Conclusion

 The detection rate of bone scan in biochemically recurrent prostate cancer patients following RP is relatively low (5%). However, the probability of positive bone scan is significantly greater in patients with serum PSA >1 ng/mL and PSADT <6 months. In Thailand, bone scans should remain the preferred option for metastatic screening due to their accessibility and cost-effectiveness.
